# COVID-19 pneumonia imaging follow-up: when and how? A proposition from ESTI and ESR

**DOI:** 10.1007/s00330-021-08317-7

**Published:** 2021-10-29

**Authors:** K. Martini, A. R. Larici, M. P. Revel, B. Ghaye, N. Sverzellati, A. P. Parkar, A. Snoeckx, N. Screaton, J. Biederer, H. Prosch, M. Silva, A. Brady, F. Gleeson, T. Frauenfelder, On behalf of the European Society of Thoracic Imaging (ESTI), the European Society of Radiology (ESR)

**Affiliations:** 1 Institute of Diagnostic and Interventional Radiology, University Hospital Zurich, Rämistrasse 100, 8091 Zurich, Switzerland; 2 Department of Diagnostic Imaging, Oncological Radiotherapy and Hematology, Fondazione Policlinico Universitario “A. Gemelli” IRCCS, Università Cattolica del Sacro Cuore, Rome, Italy; 3 Department of Radiology, Cochin Hospital, Université de Paris, Paris, France; 4 Department of Radiology, Cliniques Universitaires Saint Luc, Catholic University of Louvain, Brussels, Belgium; 5 Scienze Radiologiche, Department of Medicine and Surgery, University of Parma, Parma, Italy; 6 Department of Radiology, Haraldsplass Deaconess Hospital and Department of Clinical Medicine, Faculty of Medicine and Dentistry, University of Bergen, Bergen, Norway; 7 Department of Radiology, Antwerp University Hospital and University of Antwerp, Antwerp, Belgium; 8 Department of Radiology, Royal Papworth Hospital, Cambridge, UK; 9 Department of Diagnostic and Interventional Radiology, University Hospital of Heidelberg, Heidelberg, Germany; 10 Member of the German Lung Research Center (DZL), Translational Lung Research Center Heidelberg (TLRC), Im Neuenheimer Feld 430, 69120 Heidelberg, Germany; 11 Faculty of Medicine, University of Latvia, Raina bulvaris 19, Riga, 1586 Latvia; 12 Faculty of Medicine, Christian-Albrechts-Universität Zu Kiel, 24098 Kiel, Germany; 13 Department of Biomedical Imaging and Image-Guided Therapy, Medical University of Vienna, Vienna, Austria; 14 Department of Radiology, Mercy University Hospital, Cork, and University College Cork, Cork, Ireland; 15 Department of Oncology, University of Oxford, Oxford, UK

**Keywords:** COVID-19, Diagnostic imaging, Multidetector computed tomography, Lung, Follow-up

## Abstract

**Abstract:**

This document from the European Society of Thoracic Imaging (ESTI) and the European Society of Radiology (ESR) discusses the role of imaging in the long-term follow-up of COVID-19 patients, to define which patients may benefit from imaging, and what imaging modalities and protocols should be used. Insights into imaging features encountered on computed tomography (CT) scans and potential pitfalls are discussed and possible areas for future review and research are also included.

**Key Points:**

*• Post-COVID-19 pneumonia changes are mainly consistent with prior organizing pneumonia and are likely to disappear within 12 months of recovery from the acute infection in the majority of patients.*

*• At present, with the longest series of follow-up examinations reported not exceeding 12 months, the development of persistent or progressive fibrosis in at least some individuals cannot yet be excluded.*

*• Residual ground glass opacification may be associated with persisting bronchial dilatation and distortion, and might be termed “fibrotic-like changes” probably consistent with prior organizing pneumonia.*

**Supplementary Information:**

The online version contains supplementary material available at 10.1007/s00330-021-08317-7.

## Background

The term “long COVID” was first mentioned in the literature in June 2020 [1]. In autumn 2020, olfactory symptoms, mainly the loss of smell or altered sense of smell, were the first reported persisting symptoms, followed by recognition that symptoms could linger in all parts of the body [1]. The *British Medical Journal* guidelines define persistent symptoms after 4 weeks as long COVID, and post-COVID syndrome when symptoms last longer than 12 weeks [1]. It affects about 10% of COVID-19 patients [2, 3]. The risk of developing long COVID or post-COVID syndrome is dependent on several factors, including the severity of the primary infection, age, gender, and body mass index [3]. In addition, the need for admission to and the duration of admittance to intensive care units (ICU) and mechanical ventilation, plus possible smoking history and chronic alcoholism, have also been cited as potential risk factors for the development of persisting pulmonary abnormalities following COVID-19 pneumonia [4].

The incidence of COVID-19 pneumonia patients managed with mechanical ventilation has been reported as being between 29 and 89% [5], with the numbers varying as papers report the incidence of ventilation, with this varying across the world and changing over the course of the pandemic. Ventilator-associated pneumonia (VAP) is defined as pneumonia which occurs after 48 h of mechanical ventilation [6]. COVID-19 pneumonia patients have an increased risk of VAP, with 48% of patients developing VAP compared to 13% in non-COVID-19 pneumonia patients [7].

The risk of developing pulmonary fibrosis after acute respiratory distress syndrome (ARDS) is well known and was recognized long before COVID-19 pneumonia [8, 9]. Previous pulmonary infection resulted in fibrosis in 4% after the first severe acute respiratory syndrome (SARS) epidemic in the early 2000s [10] and up to 25% after H1N1 [11]. The rate of suspected lung fibrosis on CT in COVID-19 pneumonia patients varies between about 46% at 2 months and 25–30% at 3 months and 6 months respectively [12–14]. Longer-term follow-up studies are awaited to determine if these CT features will persist or resolve.

However, pulmonary dysfunction is not the only long-term symptom. Several other symptoms may persist and present as thromboembolic, cardiovascular, neuropsychiatric, renal, gastrointestinal, or dermatological disease [15]. The most common self-reported symptoms are fatigue, dyspnea, headache, and loss of sense of smell [3]. The purpose of this paper is to describe the role of imaging in long COVID patients and post-COVID syndrome. Clinical aspects in the follow-up of COVID-19 patients can be found in a statement (currently under review) from the European Respiratory Society (ERS).

## Role of Imaging

### What are the CT features encountered in patients after COVID-19 pneumonia?

#### CT features at discharge

The sequential pulmonary alterations during the early stage of the acute phase of COVID-19 pneumonia include peripheral and bilateral ground glass opacities (GGOs) as the predominant findings, followed, as the disease progresses, by consolidation that gradually resolves in survivors [16]. Some patients were reported to develop cystic lung disease, such as pneumatoceles. However, these changes are mostly due to barotrauma during mechanic ventilation (ventilation-associated lung injury) [17, 18]. Radiological appearances of cyst formation secondary to COVID-19 are reported, but with relatively low prevalence [19].

The rate of full resolution at patient discharge varies between studies, according to the initial severity of the infection; indeed, the full representation of imaging follow-up is not known (e.g., patients with minor signs of acute pneumonia are less likely to undergo follow-up with pulmonary imaging). Lung consolidation has been reported to show faster improvement [20]. Patients at discharge may therefore present with normal lungs or residual GGO as the most frequent pulmonary appearance on CT [20, 21].

**Fig. 1:**
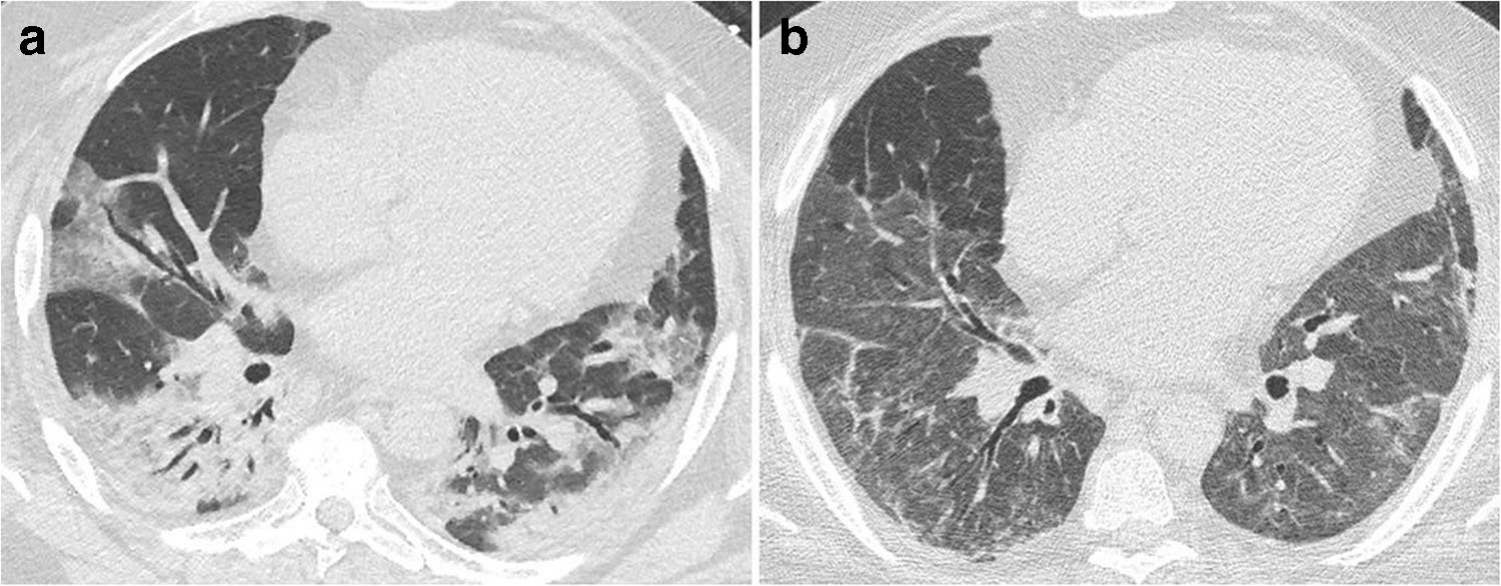
CT features at baseline and at discharge in a 52-year-old man who developed severe COVID-19 pneumonia (intubation, extracorporeal membrane oxygenation, 45 days in ICU). **a** Baseline CT showing bilateral consolidation and ground glass opacities (GGOs) with peripheral and peribronchovascular distribution. **b** 2-month CT follow-up demonstrating the complete resolution of consolidation, with residual parenchymal bands bilaterally, while GGOs appear more extensive (“tinted sign”) in the lower lobes, lingula, and right middle lobe. Note the improved expansion of the left lung in **b**

Residual disease at discharge has been reported in up to 94% of patients [22]. Interestingly, GGO may first extend further in the lungs while decreasing in attenuation, a phenomenon reported as the “tinted sign,” possibly reflecting a gradual regression of inflammation associated with re-expansion of the alveoli [12, 20] (Fig. 1). Most residual disease will further regress, and complete recovery has been reported in 25–42% at 2 weeks and 53–65% at 3 to 4 weeks after discharge [20, 21]. Consequently, imaging follow-up should be performed after a delay to allow resolution of the reversible inflammatory process. There are recommendations to perform CT follow-up at 12 weeks post discharge in patients with persistent respiratory symptoms, after clinical assessment and pulmonary function test evaluation [23].

#### CT features at three months

At 3 months, there are remaining CT abnormalities reported in 17 to 91% of discharged COVID-19 pneumonia patients [13, 24–27]. The highest range was reported in patients who were admitted to ICU [28], while it is expected that patients with minor signs of acute pneumonia might reverse in the vast majority of cases.

**Fig. 2:**
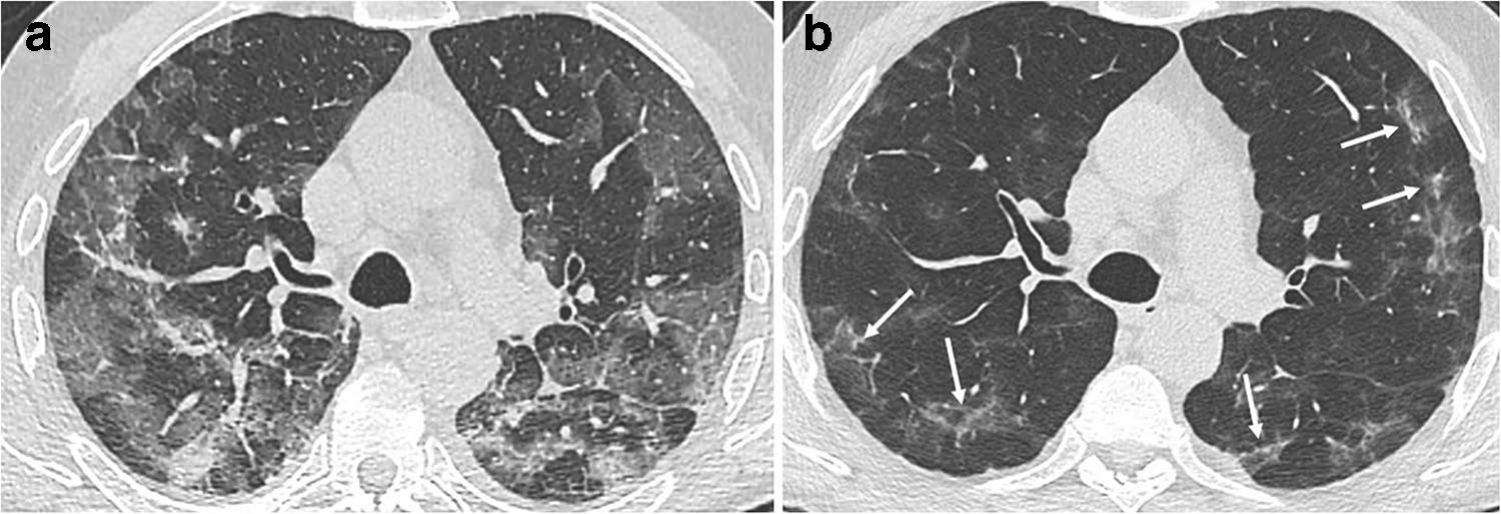
CT features at baseline and 3-month follow-up, in a 60-year-old man with initial medium severity (10-day oxygen supplementation). **a** Baseline CT showing bilateral peripheral ground glass opacities (GGOs). **b** 3-month CT follow-up. GGOs significantly improved. Some areas of linear consolidation parallel to the pleura are demonstrated (arrows), consistent with late phase organizing pneumonia. Mild deformation of the left major fissure is noted, but there are no formal signs of fibrosis such as traction bronchiectasis or honeycombing

**Fig. 3:**
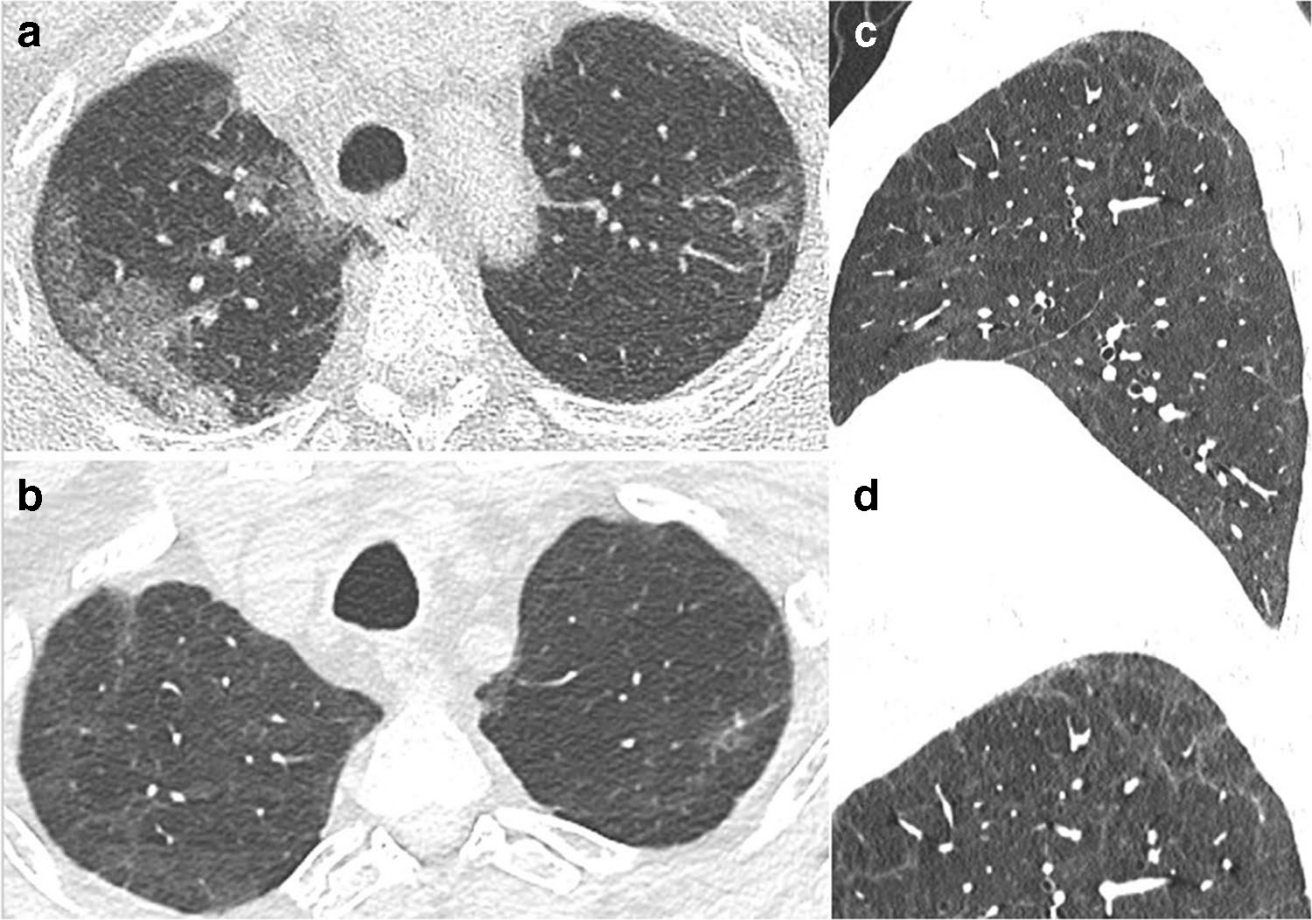
CT features at baseline, and 4-month follow-up, in a 52-year-old woman who developed severe COVID-19 pneumonia (intubation, 42 days in ICU). **a** Baseline CT. **b**, **c** 4-month CT follow-up. Ground glass opacities (GGOs) have partially resolved (low-density GGO), with remaining linear opacities on the axial transverse view through the upper lung (**b**). On the sagittal reformation (**c**), the polygonal shape suggests that linear opacities on the axial transverse view have a perilobular distribution (**d**, magnification), which might indicate late phase organizing pneumonia

**Fig. 4:**
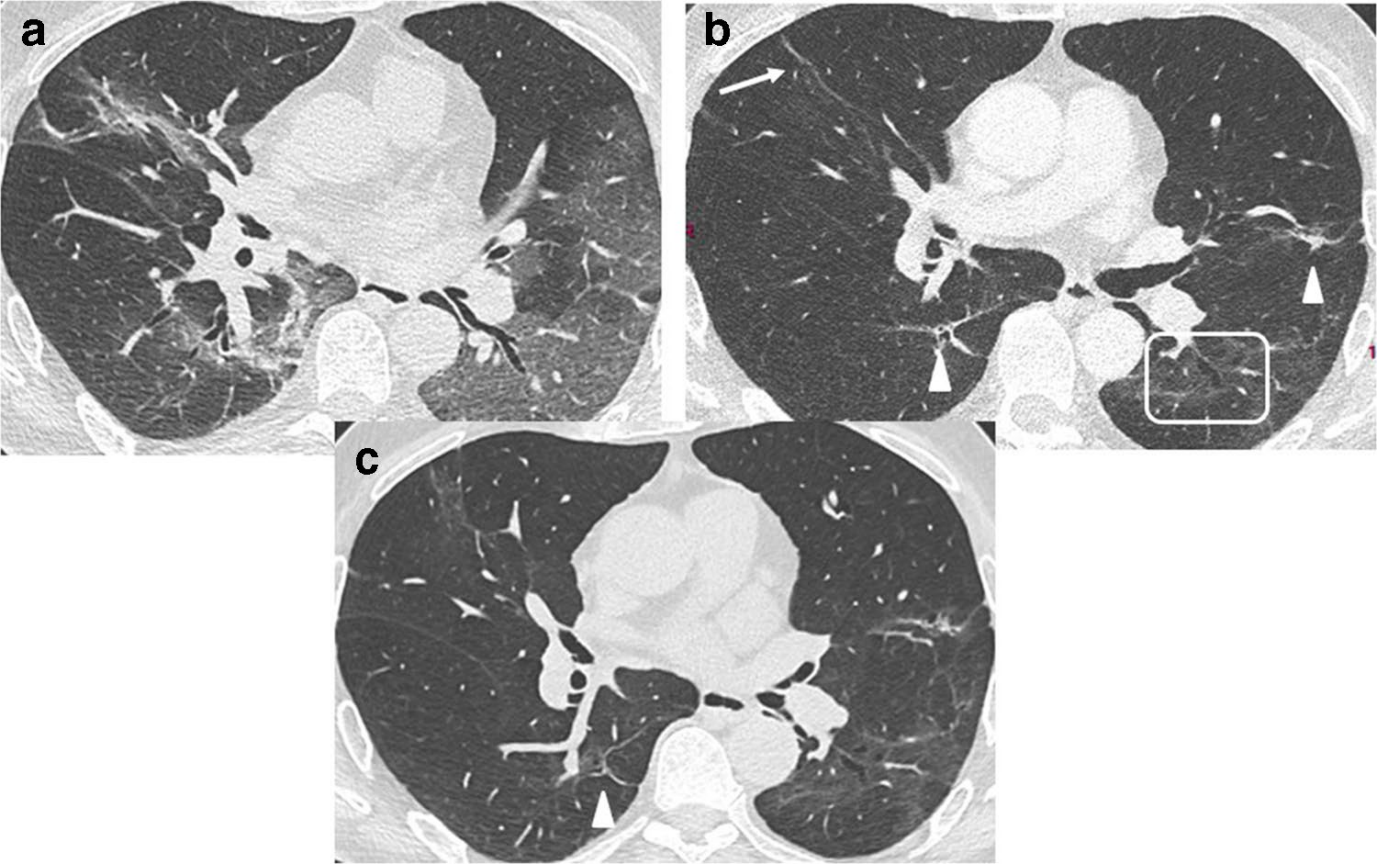
CT features at baseline and 4-month and 1-year follow-up, in a 65-year-old man who developed severe COVID-19 pneumonia (intubation, 27 days in ICU). **a** Baseline CT. **b** 4-month CT follow-up. Ground glass opacities (GGOs) have largely resolved, except in the upper segment of the left lower lobe. Bronchial dilatation (square) is demonstrated within the residual GGO area. Linear consolidation (arrow head) and parenchymal band (arrow) are also denoted. **c** 1-year follow-up. CT features are unchanged. There are no honeycombing and no peripheral reticulations

Low-attenuation GGO remains the most frequent CT finding, observed in up to two-third or more of patients [13, 24–26]. Other CT findings include linear consolidation or band-like and perilobular opacities, possibly in part consistent with organizing pneumonia, and also reticulation and interstitial thickening (Figs. 2, 3, and 4). A greater extent of CT abnormalities is associated with longer hospitalization, ICU admission, and ventilation [24, 29].

Fibrotic-like lung changes on CT such as reticulation associated with traction bronchiectasis have been reported in 21 to 26% of patients [24, 26, 28] (Fig. 4). These have been reported to be associated with ventilation and length of stay in the ICU [24, 28]. However, the term traction bronchiectasis, suggesting irreversible fibrotic changes, should be used with caution, as bronchial dilatation and distortion in areas of consolidation or GGO may be reversible. There is a risk of overestimating definite fibrotic changes, given that CT abnormalities persisting at 3 months usually improve with subsequent follow-up, especially in patients who did not experience severe forms of the disease. Honeycombing, representing the definite CT feature of pulmonary fibrosis, has been uncommonly reported and may potentially be associated with a pre-existing fibrosing lung disease [30].

**Fig. 5:**
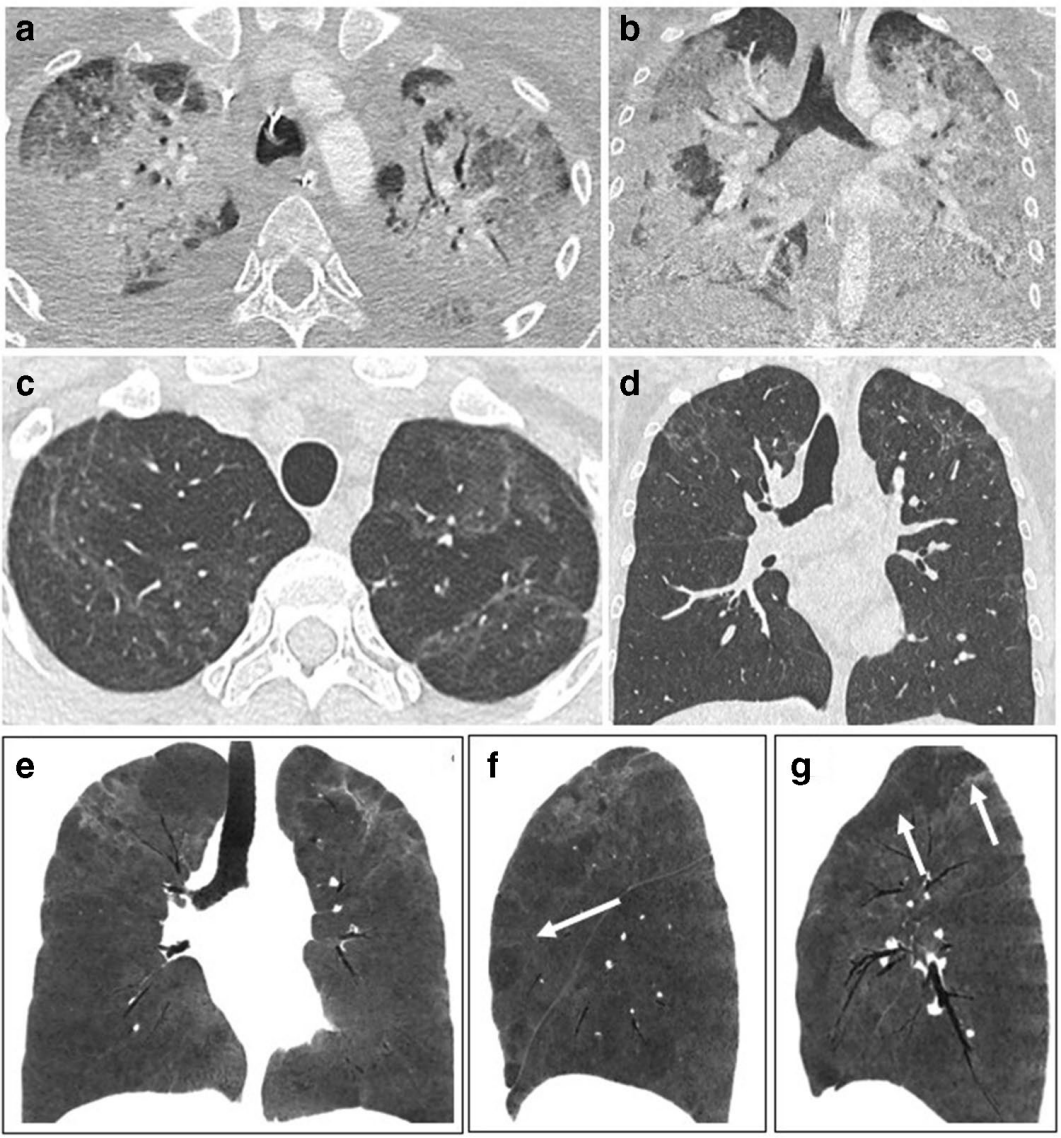
CT features at baseline, and 1-year follow-up, in a 30-year-old woman who developed severe COVID-19 pneumonia (intubation, 40 days in ICU). **a**, **b** Baseline CT showing extensive ground glass opacities (GGOs) and consolidation. **c**–**g** 1-year CT follow-up. Residual GGO, with a linear shape, is mainly seen in the upper lung, suggestive of organizing pneumonia at a late phase. There is no bronchial dilatation, signs of architectural distortion, or honeycombing, as confirmed by minimum intensity projections (minIP) (**e**, **f**, **g**). The sagittal minIP reformations (**f**, **g**) demonstrate plurilobular areas of decreased attenuation (arrows). This mosaic attenuation pattern might be due to either residual small airway disease or peripheral vascular obstruction. DLCO at 1 year was 64%

Guler et al reported a mosaic attenuation pattern with areas of hypoattenuation in 66% of patients with previous severe/critical disease, compared to 13% in patients with mild/moderate forms (Fig. 5). Whether this may be attributed to small airway disease or pulmonary vascular microthrombosis remains unclear [30, 31]. Correlation of CT features at 3 months with clinical signs such as dyspnea remains weak [24, 26].

#### CT features at longer-term follow-up

While short-term follow-up CT findings have now been largely described, there are fewer reports on longer-term follow-up appearances.

**Fig. 6:**
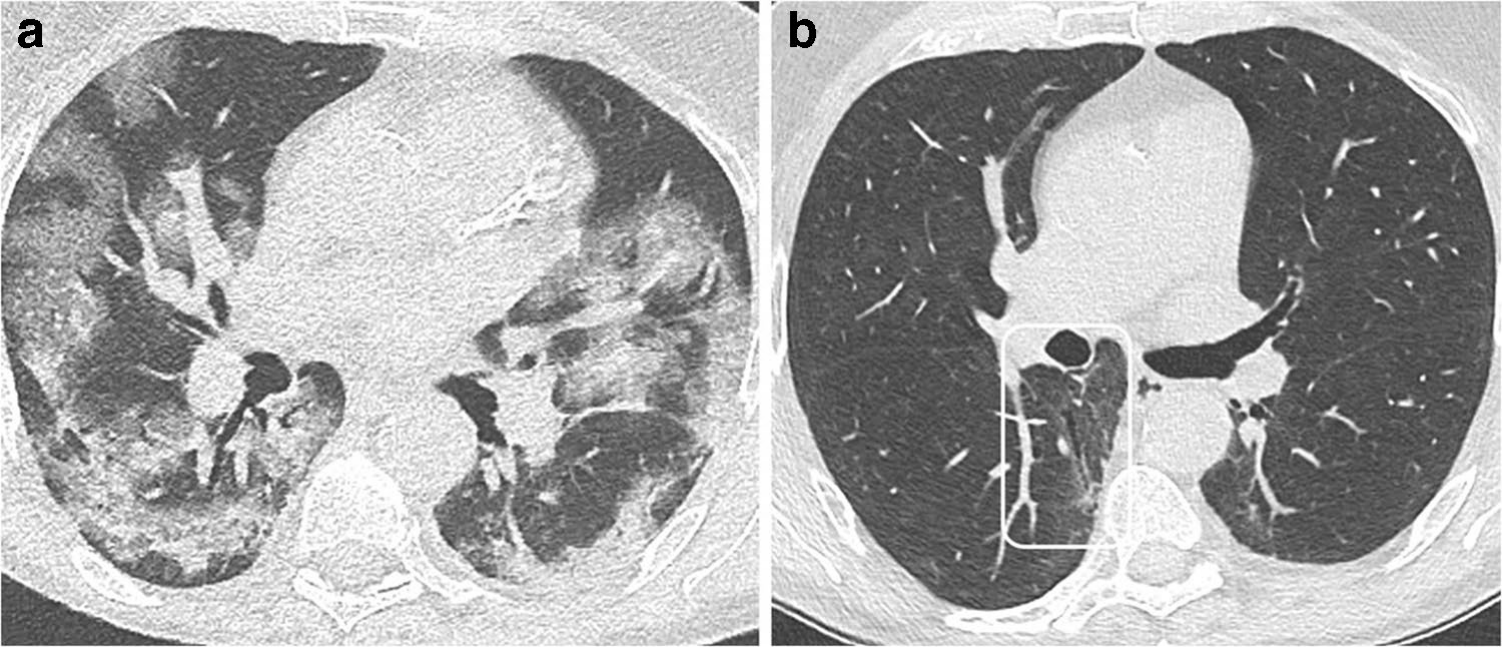
CT features at baseline, and 1-year follow-up, in a 63-year-old man who developed severe COVID-19 pneumonia (intubation, 9 days in ICU). **a** Baseline CT. **b** 1-year CT follow-up. Ground glass opacities (GGOs) have largely resolved. Mild bronchial dilatation (rectangle) is demonstrated within the residual GGO area of the right lower lobe. Pulmonary function tests are normal, with a DLCO value of 90%

Han et al reported “fibrotic-like” changes at 6-month follow-up in 35% of COVID-19 patients who had severe disease, according to the World Health Organization’s interim guidance diagnostic criteria [12]. These “fibrotic-like” changes included traction bronchiectasis (Figs. 4 and 6) and honeycombing, as well as interlobar pleural traction and thickening of the pleura, the latter two being more questionable. Honeycombing and traction bronchiectasis were respectively observed in 2 and 10% of patients at 6 months.

It is unclear whether the reported “so-called” traction bronchiectasis represents irreversible change, as it has been reported in survivors of the preceding SARS epidemic that CT abnormalities present at 6 months may show improvement at 84-month follow-up [32].

In a recent report, 27 of 41 patients (61%) evaluated at 7 months, of whom 15 had initially severe disease, achieved complete radiological resolution on the 7-month follow-up CT, with persisting bronchial distortion present in 29% [33].

It is important to be aware that in ARDS survivors, it has been reported that long-term disability is mainly due to causes other than lung fibrosis, with mild abnormalities only identified on CT performed between the 2-year and 5-year visits [34].

In keeping with these reports, the currently available longest follow-up series on 83 patients after survival of severe COVID-19 pneumonia reports a high prevalence of residual findings on CT at discharge from the hospital, with a continuous improvement 12 months after discharge [35]. In this study, the number of individuals with residual changes on CT improved from 65 (78%) at 3 months after discharge from hospital to 40 (48%) at 6 months, 22 (27%) at 9 months, and 20 (24%) at 12 months after discharge. Together with the reduction of CT abnormality prevalence, the spectrum of findings changed: typical features of COVID-19 pneumonia such as interlobular septal thickening, reticular opacity, and subpleural curvilinear opacities almost disappeared by month 9 with the predominant residual finding being ground glass opacities. Established fibrosis or progressive interstitial changes were not observed.

To summarize, most patients who were hospitalized in ICU for COVID-19 pneumonia still present with persisting CT abnormalities at 3-month follow-up. These abnormalities are probably consistent with residual post organizing pneumonia appearances and should not be described as fibrotic changes, since most are reversible and will continue to resolve up to 1 year post hospital discharge. The exact proportion of patients with long-term, irreversible fibrotic scarring is not clear, but seems to be low, probably close to 20% from the few papers published as of the time of writing, and based on what is known from the data on long-term changes seen on CT in ARDS patients. The proportion of patients with progressive fibrosis is expected to be even lower.

Importantly, there are also other causes of persistent dyspnea in COVID-19 pneumonia survivors, such as post-intubation tracheal stenosis, deconditioning, sarcopenia, and depression which may affect those who have spent long periods in ICU [34].

Figures 1, 2, 3, 4, 5, and 6 illustrate the main CT features at 3-month and 6-month follow-ups. A glossary of appropriate terms to describe the post-COVID-19 pneumonia changes is provided in Table 1 [12, 36, 37].

**Table 1:** Glossary. A proposal of a glossary of terms to describe CT findings in post-COVID-19 pneumonia based on the current literature [12, 36, 37]. Terms which are new compared to existing classifications in **bold**

Terminology	Explanation
**Low-density ground glass opacity (GGO)**	Hazy increased lung opacity that preserves bronchial and vascular margins; it represents the progressive resolution of GGO or consolidation following recovery from COVID-19 pneumonia
**Tinted sign**	GGO, which extends further into the lungs while decreasing in attenuation; it represents a possible finding following resolution of COVID-19 pneumonia and it should not be interpreted as worsening disease
Linear consolidation	Subtle consolidation which runs parallel or perpendicular to the pleura; it can be observed in the late phase of organizing pneumonia
Parenchymal bands	Stripes which run parallel or perpendicular to the pleura, consistent with late-phase organizing pneumonia
Perilobular opacities	Opacity that borders the periphery of the secondary pulmonary lobule; it can involve interlobular septa, visceral pleura, and lymphatics or veins; frequently observed in organizing pneumonia at any stage
Reticulation	Intra- and inter-lobular septal thickening; mild irregular reticulation may be seen in the late phase of COVID-19 pneumonia
Traction bronchiectasis	It is usually a sign associated with pulmonary fibrosis; it can occur in the late phase of COVID-19 pneumonia and should not be overcalled
**Bronchial distortion**	This term might be preferred over “traction bronchiectasis” when bronchial geometry is altered in the course of the disease, while its irreversibility is yet to be confirmed. This might represent an interim finding during the course of organizing pneumonia, with or without permanent sequelae. Because the terminology “traction bronchiectasis” links straight to the classic pattern(s) of irreversible pulmonary fibrosis (with discrete potential of progressive disease), the term “bronchial distortion” might take on the description of morphology distortion in the follow-up of COVID-19 pneumonia, until the irreversibility is actually confirmed. Similar lexicon might apply to fissure (e.g., fissure distortion) and vessels (e.g., vessel distortion) into the broader representation of signs of distortion
**Fibrotic-like changes**	Include reticulation associated with bronchial dilatation and distortion in areas of consolidation or GGO; these can be seen in the late phase of COVID-19 pneumonia. Fibrotic-like changes are potential precursors of fibrosis, but there is a high probability that these will resolve with time
Honeycombing	Represents the CT feature of end-stage pulmonary fibrosis; uncommonly reported in the late phase of COVID-19 pneumonia
Mosaic attenuation pattern	Abnormally hypodense areas corresponding to one or several contiguous secondary pulmonary lobules alternating with normal or abnormally hyperdense areas (i.e., GGO); it has been described as a late feature of severe COVID-19 pneumonia. It is still unclear whether it is due to residual small airway disease or microvascular thrombosis

### Who should have imaging follow-up?

In general, follow-up chest imaging in patients with community-acquired pneumonia is not routinely advised in patients whose symptoms have resolved within 5 to 7 days [38]. When follow-up imaging is performed, the rationale is to exclude an underlying malignancy, rather than to evaluate residual parenchymal findings. Predicting the respiratory consequences of COVID-19 is challenging. Experience from other coronavirus infections, including the worldwide outbreak of SARS and Middle East respiratory syndrome (MERS), suggests that the development of post-infection fibrosis is a concern [39, 40]. Research shows development of lung fibrosis and persistence of abnormal findings on chest CT scans up to 7 years after symptom presentation [32, 41]. Patients who were not hospitalized and have no clinical symptoms may not require imaging follow-up.

Taking into account the frequency of persistent lung abnormalities and the lack of data on long-term consequences, follow-up imaging should be considered in symptomatic patients who were hospitalized and/or showed a more severe clinical course of the disease. In these patients, chest CT is advised 3 months after hospital discharge. In patients who have non-resolving chest CT abnormalities, further investigations and repeated follow-up imaging after a further 3 months is advised. In all cases, imaging findings should be correlated with clinical findings and pulmonary function tests. Furthermore, chest CT imaging is advised in patients who present with new or progressive respiratory symptoms when recovering from COVID-19 pneumonia in order to assess possible complications such as pneumothorax or superimposed infection.

Pulmonary fibrosis related to the setting of ARDS is well recognized, with the majority of these patients developing histopathological evidence of fibrosis [9, 34, 42]. In view of the high prevalence of ARDS among COVID-19 pneumonia patients, the development of pulmonary fibrosis in this patient population is a concern [43, 44], and chest CT imaging in this particular group of patients is advised.

At the time of writing, specific guidelines for the long-term follow-up of COVID-19 pneumonia patients are limited. Guidelines on the respiratory follow-up of patients with a clinico-radiological diagnosis of COVID-19 pneumonia were published by the British Thoracic Society [23].

More evidence relating to this new syndrome will become available in the months to come, allowing guidelines to be updated and specific recommendations to be adjusted. Research on long-term follow-up of COVID-19 is needed to clarify the term of persistence of these lung abnormalities as well as correlation with clinical findings.

### Which imaging modalities/protocols at which disease stage should be used?

COVID-19 pneumonia is characterized by GGO as the predominant CT finding in the acute phase; moreover, some patients present with a transient increase in attenuation and the development of consolidation of variable extent and severity, and the development of linear consolidation, as a manifestation of organizing pneumonia.

Chest CT scans may be performed using a low-dose protocol, as reported by Dangis et al., who used a sub-millisievert effective radiation dose CT protocol and reported a diagnostic accuracy of 90.2%, using real-time polymerase chain reaction (RT-PCR) as the standard of reference [45]. However, extremely low-dose protocol is discouraged in the follow-up because it could hamper the perception and the confidence of subtle diffuse GGO: noise and increasing strength of iterative reconstruction are not yet explored in this pattern of post-COVID-19 sequelae [46].

As for other lung diseases, additional CT acquisition in the prone position can be useful to distinguish mild or limited COVID-19 pneumonia from normal dependent density [47].

After discharge, CT protocols should be adapted to suit the clinical question. Minimum intensity projection (minIP) reformation may be useful to better depict residual abnormalities (Fig. 5).

Expiratory CT may be helpful if there is a suspicion of residual small airway disease. Dual-energy CT might be performed in dyspneic patients with decreased diffusing capacity (DLCO) if the unenhanced inspiratory CT scan appears normal or minimally abnormal to help identify possible perfusion defects secondary to microvascular thrombosis [48].

As mentioned earlier, there are potential causes of persisting dyspnea other than lung fibrosis or microvascular thrombosis, such as post-intubation tracheal stenosis, which can be overlooked if the cervical portion of the trachea is not included on the CT scan.

To summarize, unenhanced low-dose CT performed supinely should be sufficient in the vast majority of COVID-19 patients, both in the acute phase and on follow-up. Expiratory CT and single- or dual-energy contrast-enhanced studies should only be performed on selected patients, after clinical and functional evaluation (see supplementary material).

## The role of pulmonary angiography CT and dual-energy CT

Patients infected with SARS-Cov-2 are at risk of thromboembolic complications, due to the activation of coagulation linked to the immune response. Severe COVID-19 pneumonia is associated with increased levels of inflammatory and prothrombotic biomarkers such as interleukin-6, which play a fundamental role in the activation of the extrinsic coagulation route and suppress the fibrinolytic system [49]. Furthermore, it has been demonstrated that SARS-Cov-2 has the capacity to directly infect the alveolar endothelial cells and induce endothelitis, which can also promote local thrombosis [50]. There is now an established awareness of the increased risk of pulmonary embolism (PE) from numerous articles reporting PE in COVID-19 pneumonia patients, with the consequent possible risk of the over-use of CT pulmonary angiography (CTPA) [51–59].

However, the reported incidence of PE varies widely, from less than 6% when CTPA is systematically performed at the initial presentation of unselected patients [51] to 30–38% when CTPA is performed in severely ill patients admitted to ICU [59]. A meta-analysis including 27 studies with 3342 patients reported that PE was more frequently found in severely ill patients. The pooled incidence rates were 24.7% (95% CI: 18.6, 32.1) in patients who were critically ill or who were admitted to ICU vs. 10.5% (95% CI: 5.1, 20.2) for patients who were not admitted to ICU [60]. Another meta-analysis based on 3487 patients from 30 studies reported venous thromboembolism (VTE) in 24% of patients admitted to ICU, and in 9% of patients from normal wards [61]. Even though the symptoms of COVID-19 pneumonia and pulmonary embolism strongly overlap, especially dyspnea and/or oxygen desaturation, there is no current recommendation to routinely perform a CTPA when evaluating COVID-19 pneumonia patients. The 2020 advice from the European Society of Radiology and the European Society of Thoracic Imaging was to consider performing an additional contrast-enhanced CT acquisition for patients requiring supplementary oxygen if there was limited disease extension on the unenhanced CT [16].

D-dimer elevation has been recognized as a useful biomarker of poor prognosis; D-dimer levels are now almost invariably checked in COVID-19 pneumonia patients [62]. This greatly increases the referral rate for CTPA, with a risk of over-use. The best cutoff for predicting VTE in COVID-19 pneumonia patients has still not been determined. The acceptable maximum failure rate of PE diagnostic strategies should be adapted to the prevalence of PE in the specific population, according to the recommendation of the Scientific and Standardization Committee (SSC) of the International Society of Thrombosis and Haemostasis (ISTH) [63]. In COVID-19 pneumonia outpatients or inpatients from normal wards where PE prevalence is low, using other D-dimer cutoffs as those used in the general population might expose to unacceptable failure rates, whereas higher thresholds could potentially be used for ICU patients. Mouhat et al.identified 2590 ng·mL^−1^ to be the best independent predictor of PE in their predominantly inpatient COVID-19 pneumonia population, in whom the PE prevalence was 27% [64].

Dual-energy CT (DECT) enables assessment of pulmonary perfusion and the detection of capillary microvascular thrombosis, which has been reported to occur in severe COVID-19 pneumonia patients [65]. In the acute setting, DECT has been reported to demonstrate increased perfusion in areas of GGO. The loss of physiological hypoxic vasoconstriction induces pulmonary shunting, which may explain the profound hypoxemia observed in some COVID-19 pneumonia patients [66]. DECT has also been used in patients after discharge. Diffuse hypo-perfused areas were reported in 3 patients with dyspnea on minimal exertion and no remaining parenchymal CT anomalies [48]. If validated in larger series, DECT might be used in patients with persisting abnormal gas exchange despite apparent normalization of lung parenchyma on CT. To date, there is no evidence in the literature that macro-vascular disease plays a role in long COVID or post-COVID syndrome. If there are still peripheral perfusion deficits in the late follow-up is unclear and subject to current investigation [48]. Therefore, currently, CTPA is only suggested if there is further evidence of PE following the general consideration of pre-test probability and D-dimers, especially if supplementary oxygen is needed in patients with limited disease extension [16]. Additionally, CTPA can be considered in the follow-up of patients who had PE in the acute phase of the disease.

## Conclusion

Most patients show residual disease at discharge. Post-COVID-19 pneumonia changes are mainly consistent with prior organizing pneumonia and are likely to disappear within 12 months of recovery from the acute infection in the majority of patients. “Fibrotic-like” changes are potential precursors of fibrosis, but there is a high probability that these will resolve over time. Consequently, imaging follow-up should be performed after a delay to allow resolution of the reversible inflammatory process in symptomatic patients. Unenhanced low-dose CT performed supinely should be sufficient in the vast majority of COVID-19 patients for follow-up. Expiratory CT and single- or dual-energy contrast-enhanced studies should only be performed on selected patients, after clinical and functional evaluation.

## Supplementary Information

Below is the link to the electronic supplementary material.

Supplementary file1 (DOCX 131 KB)

## Abbreviations

AI: Artificial intelligence
ARDS: Acute respiratory distress syndrome
COVID-19: Corona virus 2019
CT: Computed tomography
CTPE: CT pulmonary angiography
DECT: Dual-energy computed tomography
DLCO: Diffusing capacity
ERS: European Respiratory Society
ESR: European Society of Radiology
ESTI: European Society of Thoracic Imaging
GGOs: Ground glass opacities
ICU: Intensive care units
ISTH: International Society of Thrombosis and Haemostasis
MERS: Middle East respiratory syndrome
minIP: Minimum intensity projection
PE: Pulmonary embolism
PET: Positron emission tomography
RT-PCR: Real-time polymerase chain reaction
SARS: Severe acute respiratory syndrome
SSC: Scientific and Standardization Committee
VAP: Ventilator-associated pneumonia
VTE: Venous thromboembolism

## Declarations

### Guarantor

The scientific guarantor of this publication is Prof. Thomas Frauenfelder.

### Statistics and biometry

No complex statistical methods were necessary for this paper.

### Methodology

• Special report
