# Supplementary material for: COVID-19 pneumonia imaging follow-up: when and how? A proposition from ESTI and ESR
Source: Eur Radiol. 2021 Oct 29;32(4):2639–49. doi: 10.1007/s00330-021-08317-7 (PMC8553396; doi:10.1007/s00330-021-08317-7)
Supplement: Supplementary file 1 — Supplementary file1 (DOCX 131 KB) [file 330_2021_8317_MOESM1_ESM.docx]

**Supplementary material**

**1. Overview**

There is increasing evidence from the global COVID-19 pandemic that some patients suffer from prolonged multiorgan symptoms and complications beyond the initial period of acute infection. The symptoms described by patients include chronic cough, shortness of breath, chest tightness, cognitive dysfunction, and extreme fatigue [1, 2]. These findings have led to the description of a post-COVID syndrome [1, 3]. In recent months, several studies have described persisting pulmonary changes on CT in patients with post-COVID syndrome [4–6]. New terms such as "fibrotic-like" changes have been described without precise patho-physiological knowledge of their causality [4, 7]. Subtle changes in perfusion and ventilation have also been described in patients with symptoms [8]. Additionally, as vaccination has become more widespread, imaging has identified large volume of metabolically FDG avid reactive lymphadenopathy which may cause diagnostic difficulties [9].

**2. Artificial intelligence: critical appraisal and perspective for clinical use in follow-up**

Artificial intelligence (AI) algorithms have been developed for use in COVID-19 pneumonia, yet its clinical utility is still debated and unproven [10, 11]. Among the possible uses of AI in COVID-19 pneumonia [12], there are three major domains: diagnosis, prognosis, and long-term follow-up. Over the last year, a number of radiomics and AI-based models for the diagnosis and early prognostication of COVID-19 pneumonia using CT as well as CXR have been published: several approaches achieved performance similar to that of expert chest radiologists, with easy scalability to general practice [13–17]. However, many of the publications evaluating these models have suffered from a variety of methodological problems [10, 13–17] and the additional value of AI to current clinical practice appears limited, as most of the algorithms did not significantly improve current clinical prediction models [18].

The use of AI for long-term follow-up is now being explored [19] and should be driven by the experience from this first year of a “gold rush” in thoracic imaging of COVID-19, as well as the consolidated knowledge from “traditional chronic ILDs” [20]. The real value of AI in COVID-19 pneumonia potentially is in the objective classification and quantification of parenchymal abnormalities [20] as well as the prediction of longitudinal behavior [7]. Nonetheless, post-COVID syndrome requires integrated rather than organ or disease specific approaches [21]. Besides the analysis of the lung parenchyma, AI offers the possibility to further investigate pulmonary vascular changes and to combine imaging and clinical data. This holistic development and use of AI algorithms in post COVID-19 patients may help in determining the actual value of imaging compared to other tests performed for follow-up such as pulmonary function tests and diffusing lung capacity.

**3. Potential pitfalls**

A first report on findings 12 months after hospital discharge is encouraging and suggests that abnormalities identified on CT continue to improve over 12 months [22]. We therefore suggest using the term “fibrotic-like changes”, which has been used to communicate that there is a high probability that these will resolve with time, but that follow-up is warranted [4, 7]. Additionally, the high frequency of non-invasive mechanical ventilation makes it uncertain if fibrotic changes after mechanical ventilation are associated with pneumonia or reflect ventilator-induced lung injury [23].

Another potential pitfall in COVID-19 is related to vaccination: caution has to be used in staging and follow-up of cancer patients after COVID-19 vaccination. FDG avid and enlarged reactive ipsilateral lymphadenopathy identified on Positron emission tomography (PET)-CT scans after vaccination is well known [24]. However, due to the world-wide mass COVID-19 vaccination programs a greater percentage of patients is being vaccinated than would usually be the case, and it is consequently likely that some of the recently-vaccinated population will undergo imaging studies for malignancy. There are already multiple reports of FDG avid enlarged axillary lymphadenopathy identified on PET-CT scans ipsilateral to the vaccinated site [9, 25–27]. To minimize false and equivocal reports in oncological patients, whenever possible imaging follow-up for oncologic disease should be scheduled with an appropriate time interval after vaccination, and if appropriate the vaccine injection site should be contralateral to the relevant site of tumor involvement [27]. It is essential to document the vaccination status and site before imaging, and for the radiologist to have this information available at the time of reporting. If axillary lymphadenopathy ipsilateral to the vaccinated arm is identified and of diagnostic concern, it may be appropriate to perform a follow-up axillary ultrasound at 4 weeks rather than proceeding directly to node biopsy [25].

**Fig. 1 Supplementary material – Imaging follow-up in COVID-19 patients.** Intensive care unit (ICU), Computed tomography (CT), Pulmonary embolism (PE), Contrast enhanced (CE), Computed tomography pulmonary angiography (CTPA).


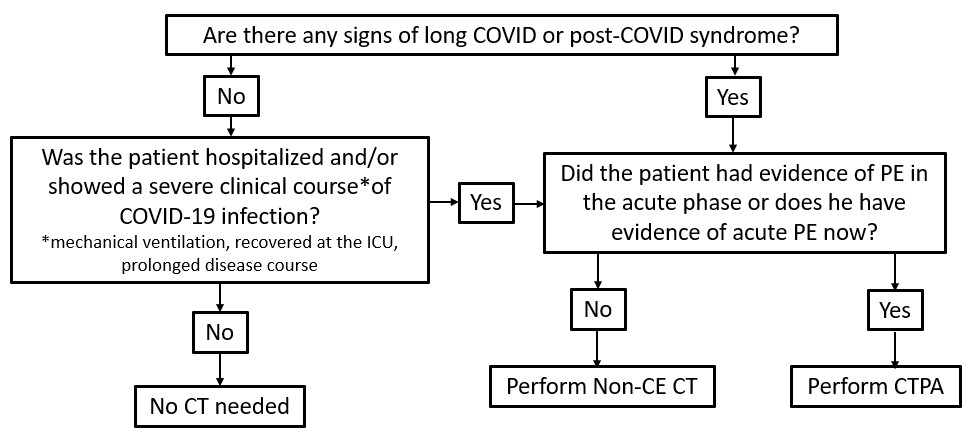


**References:**

1. Mandal S, Barnett J, Brill SE, et al (2020) “Long-COVID”: a cross-sectional study of persisting symptoms, biomarker and imaging abnormalities following hospitalisation for COVID-19. Thorax. https://doi.org/10.1136/thoraxjnl-2020-215818

2. Cordasco EM, Burns DE, Beerel F, et al (1995) Noncardiac pulmonary edema, newer environmental aspects. An update. Angiology 46:759–766. https://doi.org/10.1177/000331979504600901

3. Mahase E (2020) Covid-19: What do we know about “long covid”? BMJ 370:m2815. https://doi.org/10.1136/bmj.m2815

4. Han X, Fan Y, Alwalid O, et al (2021) Six-month Follow-up Chest CT Findings after Severe COVID-19 Pneumonia. Radiology 299:E177–E186. https://doi.org/10.1148/radiol.2021203153

5. Wang Y, Dong C, Hu Y, et al (2020) Temporal Changes of CT Findings in 90 Patients with COVID-19 Pneumonia: A Longitudinal Study. Radiology 296:E55–E64. https://doi.org/10.1148/radiol.2020200843

6. Zhao F, Zheng L, Shan F, et al (2021) Evaluation of pulmonary ventilation in COVID-19 patients using oxygen-enhanced three-dimensional ultrashort echo time MRI: a preliminary study. Clin Radiol 76:391.e33-391.e41. https://doi.org/10.1016/j.crad.2021.02.008

7. Wells AU, Devaraj A, Desai SR (2021) Interstitial Lung Disease after COVID-19 Infection: A Catalog of Uncertainties. Radiology 299:E216–E218. https://doi.org/10.1148/radiol.2021204482

8. Remy-Jardin M, Duthoit L, Perez T, et al (2021) Assessment of pulmonary arterial circulation 3 months after hospitalization for SARS-CoV-2 pneumonia: Dual-energy CT (DECT) angiographic study in 55 patients. EClinicalMedicine 34:100778. https://doi.org/10.1016/j.eclinm.2021.100778

9. Özütemiz C, Krystosek LA, Church AL, et al (2021) Lymphadenopathy in COVID-19 Vaccine Recipients: Diagnostic Dilemma in Oncology Patients. Radiology 210275. https://doi.org/10.1148/radiol.2021210275

10. Roberts M, Driggs D, Thorpe M, et al (2021) Common pitfalls and recommendations for using machine learning to detect and prognosticate for COVID-19 using chest radiographs and CT scans. Nature Machine Intelligence 3:199–217. https://doi.org/10.1038/s42256-021-00307-0

11. Group BMJP (2020) Seven days in medicine: 8-14 Jan 2020. BMJ 368:m132. https://doi.org/10.1136/bmj.m132

12. Islam N, Ebrahimzadeh S, Salameh J-P, et al (2021) Thoracic imaging tests for the diagnosis of COVID‐19. Cochrane Database of Systematic Reviews. https://doi.org/10.1002/14651858.CD013639.pub4

13. Colombi D, Bodini FC, Petrini M, et al (2020) Well-aerated Lung on Admitting Chest CT to Predict Adverse Outcome in COVID-19 Pneumonia. Radiology 296:E86–E96. https://doi.org/10.1148/radiol.2020201433

14. Chassagnon G, Vakalopoulou M, Battistella E, et al (2021) AI-driven quantification, staging and outcome prediction of COVID-19 pneumonia. Medical Image Analysis 67:101860. https://doi.org/10.1016/j.media.2020.101860

15. Schalekamp S, Huisman M, van Dijk RA, et al (2020) Model-based Prediction of Critical Illness in Hospitalized Patients with COVID-19. Radiology 298:E46–E54. https://doi.org/10.1148/radiol.2020202723

16. Wang S, Zha Y, Li W, et al (2020) A fully automatic deep learning system for COVID-19 diagnostic and prognostic analysis. European Respiratory Journal 56:. https://doi.org/10.1183/13993003.00775-2020

17. Harmon SA, Sanford TH, Xu S, et al (2020) Artificial intelligence for the detection of COVID-19 pneumonia on chest CT using multinational datasets. Nature Communications 11:4080. https://doi.org/10.1038/s41467-020-17971-2

18. Lassau N, Ammari S, Chouzenoux E, et al (2021) Integrating deep learning CT-scan model, biological and clinical variables to predict severity of COVID-19 patients. Nature Communications 12:634. https://doi.org/10.1038/s41467-020-20657-4

19. Kassin MT, Varble N, Blain M, et al (2021) Generalized chest CT and lab curves throughout the course of COVID-19. Sci Rep 11:6940. https://doi.org/10.1038/s41598-021-85694-5

20. Wu X, Kim GH, Salisbury ML, et al (2019) Computed Tomographic Biomarkers in Idiopathic Pulmonary Fibrosis. The Future of Quantitative Analysis. Am J Respir Crit Care Med 199:12–21. https://doi.org/10.1164/rccm.201803-0444PP

21. Ayoubkhani D, Khunti K, Nafilyan V, et al (2021) Post-covid syndrome in individuals admitted to hospital with covid-19: retrospective cohort study. BMJ 372:n693. https://doi.org/10.1136/bmj.n693

22. Wu X, Liu X, Zhou Y, et al (2021) 3-month, 6-month, 9-month, and 12-month respiratory outcomes in patients following COVID-19-related hospitalisation: a prospective study. Lancet Respir Med. https://doi.org/10.1016/S2213-2600(21)00174-0

23. Desai SR, Wells AU, Rubens MB, et al (1999) Acute respiratory distress syndrome: CT abnormalities at long-term follow-up. Radiology 210:29–35. https://doi.org/10.1148/radiology.210.1.r99ja2629

24. Bannister S, Sudbury E, Villanueva P, et al (2021) The safety of BCG revaccination: A systematic review. Vaccine 39:2736–2745. https://doi.org/10.1016/j.vaccine.2020.08.016

25. Brown AH, Shah S, Groves AM, et al (2021) The Challenge of Staging Breast Cancer With PET/CT in the Era of COVID Vaccination. Clin Nucl Med. https://doi.org/10.1097/RLU.0000000000003683

26. Hiller N, Goldberg SN, Cohen-Cymberknoh M, et al (2021) Lymphadenopathy Associated With the COVID-19 Vaccine. Cureus 13:e13524. https://doi.org/10.7759/cureus.13524

27. Cohen D, Krauthammer SH, Wolf I, Even-Sapir E (2021) Hypermetabolic lymphadenopathy following administration of BNT162b2 mRNA Covid-19 vaccine: incidence assessed by [18F]FDG PET-CT and relevance to study interpretation. Eur J Nucl Med Mol Imaging. https://doi.org/10.1007/s00259-021-05314-2
